# An analysis of reporting quality of prospective studies examining community antibiotic use and resistance

**DOI:** 10.1186/s13063-018-3040-6

**Published:** 2018-11-27

**Authors:** Mina Bakhit, Chris Del Mar, Anna Mae Scott, Tammy Hoffmann

**Affiliations:** Centre for Research in Evidence-Based Practice (CREBP), Faculty of Health Sciences and Medicine, Bond University, Robina, QLD 4229 Australia

**Keywords:** Antibiotic resistance, Quality of reporting, Primary care

## Abstract

**Background:**

Antibiotic resistance is a global problem, but the relationship between antibiotic use and resistance development and decay is not well understood. This knowledge is best provided by prospective studies, but to be useful they must be both conducted and reported well. Little is known about the reporting quality of these studies. This study aimed to assess the quality of reporting in prospective studies that investigated antibiotic resistance following antibiotic exposure in community-based individuals.

**Methods:**

The quality of reporting of prospective studies (17 randomised trials, eight cohort studies) identified in a systematic review of the relationship between antibiotic use and resistance were assessed independently by two researchers using checklists (one for trials, one for cohort studies) developed from existing reporting guidelines for these designs and this field.

**Results:**

The mean percentage (SD, minimum-maximum) of mandatory items that were adequately described by the included studies was 59% for trials (14%, 36–84%) and 52% for cohort studies (17%, 13–70%). Most studies adequately described the study background and rationale, the type, combination, and duration of the antibiotic intervention, and the sampling procedures followed to isolate resistant bacteria. Most studies did not report the incident numbers of resistant and susceptible isolates analysed at each time point. Blinding and sample size calculation was inadequately reported in almost half of the trials and all cohort studies.

**Conclusions:**

The quality of reporting in prospective studies investigating the association between antibiotic exposure in the community and isolation of resistance isolates is variable. Some details were missing in more than half of the studies, which precludes a complete risk of bias assessment and accurate interpretation and synthesis of results.

**Electronic supplementary material:**

The online version of this article (10.1186/s13063-018-3040-6) contains supplementary material, which is available to authorized users.

## Background

Antibiotic resistance is a global public health concern, threatening lives by jeopardising successful treatment of a vast range of bacterial infections [1, 2]. It is estimated that 10 million people may die in 2050 because of resistance [3]. Antibiotic prescribing levels in primary care are high [4, 5], even though for many of the conditions for which they are prescribed (such as acute respiratory infections), antibiotics provide minimal benefits and these may not be outweighed by the harms of their use [6–10].

Antibiotic use drives resistance [11–13] and there is some indication that resistance decays over time [13]. Knowledge about the association between antibiotic use, the development of resistance, and timeframes of potential decay is important for informing public health messages, antibiotic resistance campaigns, and clinician training. However, evidence syntheses investigating the relationship between antibiotic exposure and the development and decay of resistance have been limited to two systematic reviews that have included mostly studies with retrospective designs [11, 12]. Understanding the association between antibiotic exposure and isolation of resistance bacteria is best informed by prospective study designs. Such designs offer better opportunities to control for confounding factors, including more precise timeframes of the duration between antibiotic exposure and isolation of resistance bacteria, which helps to avoid the uncertainty that is implicit in ‘time-until’ periods that are dictated by retrospective designs. As global concern about resistance increases, an increasing number of prospective studies investigating this issue are being conducted.

However, to provide interpretable evidence and enable complete risk of bias assessment, these studies need to be reported clearly and comprehensively. There are characteristics of studies that measure antibiotic exposure and resistance that are not adequately captured by existing reporting checklists and researchers may not have adequate awareness of these issues and guidance about how to report such studies. We are not aware of any studies that have examined the quality of reporting of prospective studies about antibiotic use and resistance. We aimed to assess the completeness of reporting of prospective primary studies that examined antibiotic use and resistance.

## Method

### Selection of included studies

Studies were included in this study if they had been identified as part of a recently published systematic review that assessed the extent of bacterial resistance in individuals caused by antibiotic use in primary care, and the rate of decay of resistance [13].

Full details of the systematic review search are available elsewhere [13]. Briefly, we searched PubMed, EMBASE, and the Cochrane Central Register of Controlled Trials from inception until the first week of May 2017, using MeSH terms, keywords, and forward and backward citation searches. We included randomised controlled trials and prospective cohort studies that compared antibiotic-exposed patients in the community against controls. Our outcome was the prevalence of resistance bacteria over time.

### Assessment of the quality of reporting of included studies

For the present study, we developed two assessment checklists (one for trials and one for prospective cohort studies), based on existing reporting guidelines relevant to these study designs and this field. For trials, the relevant guideline was the Consolidated Standards of Reporting Trials (CONSORT) statement [14]; for cohort studies, the relevant guidelines were Strengthening the Reporting of Observational studies in Epidemiology (STROBE) [15] and its extension for optimising reporting of epidemiological studies in Antimicrobial Stewardship (STROBE-AMS) [16]. Additional reporting recommendations that are relevant to both study types include the Template for Intervention Description and Replication (TIDieR [17]) items and those specific to resistance reporting in systematic reviews of antibiotic interventions [18].

Informed by our experience from completing the systematic review, several items necessary for the accurate interpretation of results were added to the checklists. The resultant checklists were piloted with four epidemiologists and four antibiotic resistance researchers who used them to assess the quality of reporting of a small number of eligible studies. Following this, minor modifications were made to the grouping of the items, and the explanatory wording of some of the additional items. The final version of trial checklist had 89 items and the cohort checklist had 81 items.

Two researchers (MB and AMS) then used the checklists to independently assess the quality of reporting of each included study. Each item was rated ‘Yes’ (if the study adequately described the item), ‘No’ (if it did not), or ‘Not applicable’. Agreement between assessors was reached through discussion after small batches of five studies were rated, and discrepancies resolved through discussion with a third researcher (TH or CDM). We did not calculate the agreement between the two assessors. The complete checklists with item descriptions and the source of each item are available in Additional files 1 and 2.

### Data analysis

Data were analysed using Microsoft Excel® 2016 (Microsoft, Redmond, WA, USA) and descriptive statistics were calculated.

## Results

The sample of articles consisted of 17 randomised controlled trials and eight prospective cohort studies. The trials primarily assessed the risk of isolation of post-treatment carriage of resistant bacteria. They were published from 1982 to 2016 and conducted in ten countries. The cohort studies assessed changes in resistance patterns before and after antibiotic use. They were published from 1988 to 2008 and from seven countries (the full list of included articles is shown in Additional file 3).

### Completeness of reporting - trials

**Fig. 1:**
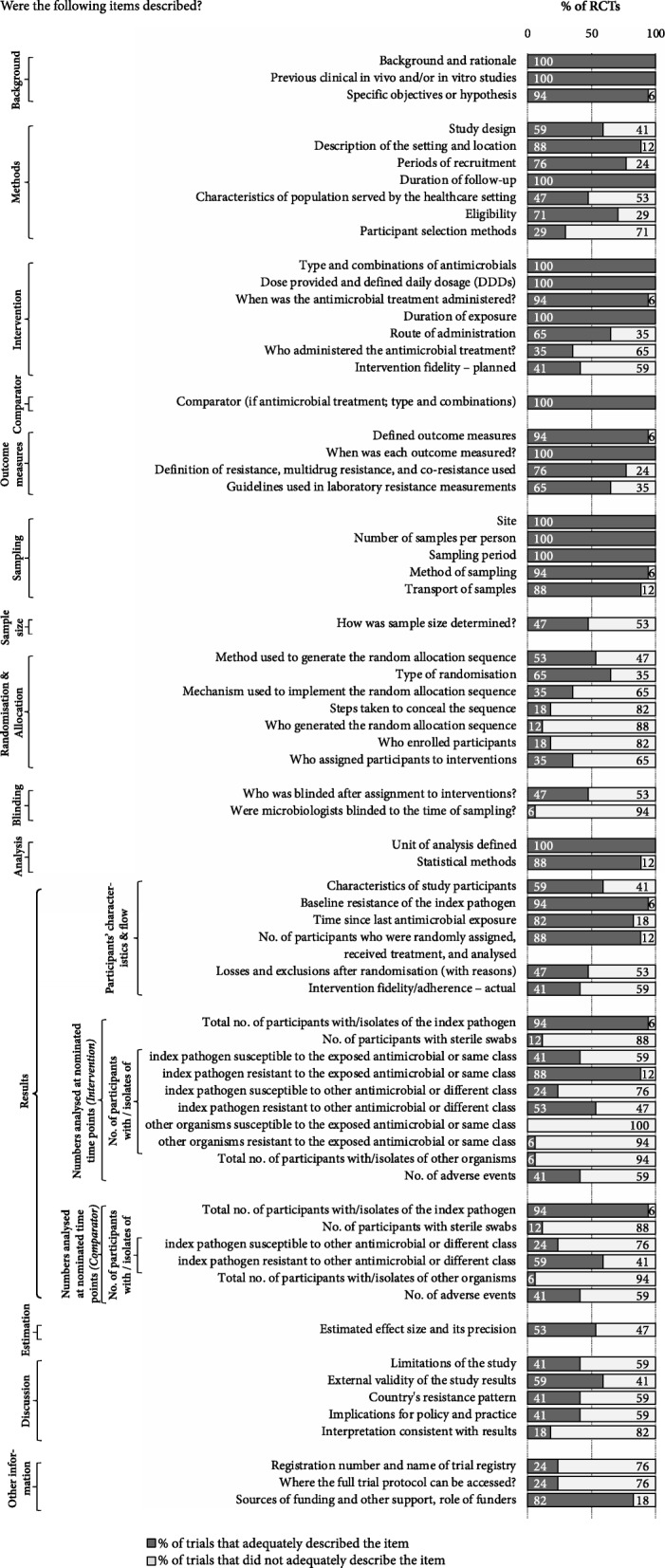
Quality of reporting, percentage of RCTs meeting each item (studies = 17, mandatory items = 70). *RCT* randomised comtrolled trial

For the 17 trials, 70 mandatory items and an additional 19 ‘if applicable’ items were scored. Twelve (17%) mandatory items were reported by all trials; one item (describing other organisms susceptible to the exposed antimicrobial or same class) was not reported by any trials. The mean percentage (SD, minimum-maximum) of the mandatory items that were adequately described by the trials was 59% (14%, 36–84%) (see Fig. 1). Additional file 4 shows the percentages of trials that adequately described each item including the ‘if applicable’ items and Additional file 5 shows the percentage of items adequately described by each trial.

The items that were most commonly reported include those about: study background (all trials provided the study rationale and described any previous in vivo and/or in vitro studies); intervention (most trials adequately described the type of antibiotics, duration and dose); and sampling (almost all trials adequately reported the sampling procedures followed for isolating resistant isolates including the site, number of samples per person, and sampling period).

Items that were poorly reported by many trials include those describing the sample size, randomisation, blinding, and the results. Almost half of the trials reported the sample size determination, and more than half of the trials poorly described the methods used for randomisation and allocation (including the person(s) responsible for generating the random allocation sequence, and the steps taken to conceal the randomisation sequence). Blinding reporting was incomplete in almost half of the trials and only one study reported blinding of microbiologists to the time of sampling. Many items describing the results were missing in the majority of the trials, including key details such as: the numbers analysed at nominated time points, particularly the number of isolates susceptible to the intervention or comparator (if applicable); and the number of participants with sterile swabs (clean-catch swabs) or resistance/susceptibility among other organisms (other than the index pathogen) isolated from other body site (other than the system/site of interest).

### Completeness of reporting - cohort studies

**Fig. 2:**
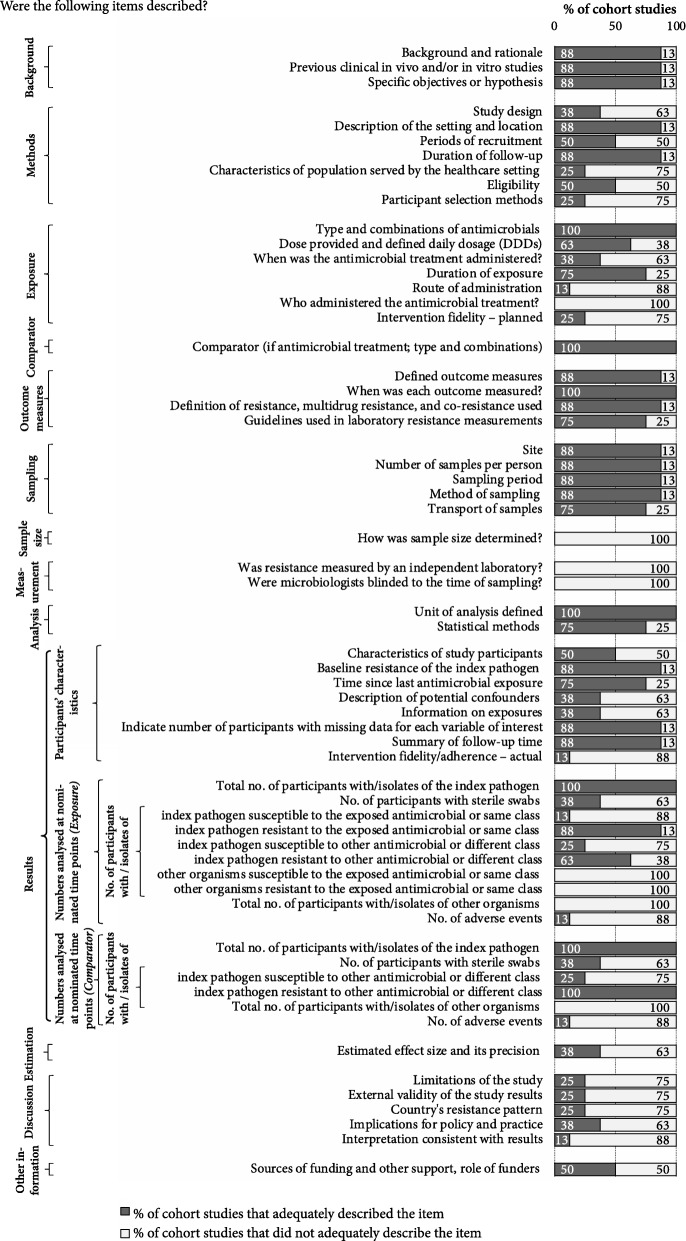
Quality of reporting, percentage of cohort studies meeting each item (studies = 8, mandatory items = 63)

In the eight cohort studies, 63 mandatory items and an additional 19 ‘if applicable’ items were scored: seven (11%) mandatory items were reported by all cohort studies; eight (13%) were not reported by any. The mean percentage (SD, minimum-maximum) of the mandatory items that were adequately described by the studies was 52% (17%, 13– 70%) (see Fig. 2). Additional file 6 shows the percentage of cohort studies that adequately described each item including the ‘if applicable’ items and Additional file 7 shows the percentage of items adequately described by each study.

The items most commonly reported were those about the: study background (most described specific objectives and study rationale); outcome measures (most defined each measure and reported when each was measured); and sampling (most studies adequately described the sampling procedures used, including the site, number of samples per person, and sampling period).

Items that were poorly reported were those describing the sample size, measurement and results. None of the studies reported how their sample size was determined and none reported if resistance was measured by an independent laboratory or if the microbiologists were blinded. As with the trials, many key details about the numbers analysed were missing, such as the number of susceptible isolates to antibiotic exposure analysed at each of the nominated time points, along with the resistance/susceptibility among other organisms isolated from another body site.

## Discussion

The quality of reporting of prospective studies examining antibiotic use and resistance varied in this study. Some aspects of the studies (such as the sampling procedures used and rationale for the study) were described in most, but some details were missing in many studies. Some of the missing items, such as those about blinding or the numbers analysed, are particularly important for assessing a study’s risk of bias and interpreting its results accurately.

Few studies reported the incident numbers of both resistant and susceptible isolates analysed at each time point (and for both the intervention and/or comparator groups), and only one study reported the isolation of resistant isolates from body sites other than the target site/system. This may be as important to know as the resistance in the originally infected site, from the point of view of antibiotic resistance generation in the microbiome, and hence in the community at large. Similarly, most studies only reported resistance to the class of antibiotic used, although some studies also reported resistance to other antibiotic classes – a possibly important omission because of induced co-resistance to antibiotics from different classes [19].

Reporting of how and whether blinding occurred was inadequate in almost half of the studies. Only one study reported whether the microbiologist was blinded to the time of sampling. Data on changes in resistance over time could be biased if those responsible for measuring resistance are not blinded to the time of sampling. As with other aspects of methods reporting, if a study does not describe a process, a reader cannot be sure if the process did not occur or was just not reported. This uncertainty impedes risk of bias assessment and decreases the confidence in the reported results.

While describing a study with sufficient detail to enable replication and interpretation of results is good scientific practice, many authors are not aware of all the details that need to be reported to sufficiently describe a study. To assist authors with comprehensively describing studies, reporting guidelines have been developed. However, for our sample of included studies, the impact of reporting guidelines would have been minimal, as most (88%) of the included cohort studies were published before the release of the STROBE reporting guidelines (and all before the STROBE-AMS publication), and all but one of the included trials were published before the 2010 CONSORT statement, although most (except three) were published before the 1996 CONSORT statement [20].

As our sample of studies is limited to those included in a systematic review of antibiotic resistance in individuals who were prescribed antibiotics in primary care, this may limit the generalisability of results beyond this setting. Additionally, the checklists used to assess the studies were modified from existing checklists and informed by the pragmatic experience of researchers assessing and synthesising these types of studies – the modified checklist have not been formally assessed. However, a strength of this study is the independent assessment of the included studies by two authors.

## Conclusions

In this study of the reporting quality of prospective studies examining antibiotic use and resistance, just over half of the mandatory checklist items were adequately described for the randomised trials and cohort studies included. Some items (such as the type, combination, and duration of the antibiotic intervention, and the sampling procedures used to isolate resistant bacteria) were adequately described by most studies, whereas other details (such as the incident numbers of resistant and susceptible isolates analysed at each time point) were not described by most. Improving the quality of reporting of future studies that measure antibiotic resistance is necessary to aid accurate synthesis and interpretation of results. Better reporting may be facilitated by a reporting checklist, which is created following the recommendations for developing reporting guidelines [21], that is, specific to prospective studies of antibiotic use and resistance. This will help to improve the quality of reporting available to the research community, clinicians, and policy makers.

## Additional files

Additional file 1: Checklist used to assess the RCTs and source of each item. (PDF 54 kb)

Additional file 2: Checklist used to assess the cohort studies and source of each item. (PDF 51 kb)

Additional file 3: List of studies included in the systematic review and assessed for their quality. (PDF 122 kb)

Additional file 4: Quality of reporting, percentage of RCTs meeting each item including the ‘if applicable’ items. (PDF 128 kb)

Additional file 5: Quality of reporting, percentage of items described by each trial. (PDF 29 kb)

Additional file 6: Quality of reporting, percentage of cohort studies meeting each item including the ‘if applicable’ items. (PDF 127 kb)

Additional file 7: Quality of reporting, percentage of items described by each cohort study. (PDF 31 kb)

## Abbreviations

AMS: Antimicrobial stewardship
CONSORT: Consolidated Standards of Reporting Trials statement
STROBE: Strengthening the Reporting of Observational studies in Epidemiology
TIDieR: Template for Intervention Description and Replication
