# Supplementary material for: An analysis of reporting quality of prospective studies examining community antibiotic use and resistance
Source: Trials. 2018 Nov 27;19:656. doi: 10.1186/s13063-018-3040-6 (PMC6258384; doi:10.1186/s13063-018-3040-6)
Supplement: Supplementary file 2 — Checklist used to assess the cohort studies and source of each item. (PDF 51 kb) [file 13063_2018_3040_MOESM2_ESM.pdf]

Additional file 2. Checklist used to assess Cohort studies and source of each item

Were the following items described?		Source of item
**Background**		
	Background and explanation of rationale and theory	STROBE/CONSORT
	Reported previous clinical ***in vivo*** and/or ***in vitro*** studies	STROBE-AMS
	Specific ***objectives*** or ***hypothesis***	STROBE/CONSORT
**Methods**		
	Study design ***described***	STROBE/CONSORT
	Description of the ***setting*** (e.g. hospital, Emergency department, etc.) and ***location*** (e.g. city, region, country)	STROBE-AMS/STROBE/CONSORT
	Periods of ***recruitment***	STROBE
	Duration of ***follow-up***	STROBE
	Characteristics of ***population served*** by the healthcare setting where patients were recruited e.g. urban/rural, low socioeconomic status	STROBE-AMS
	Eligibility criteria (e.g. inclusion & exclusion criteria)	STROBE/CONSORT
	Participant ***selection methods***	STROBE
Description of exposure	Type and combinations of antimicrobials ***(What)*** (e.g. Amoxicillin, Amoxicillin-clavulanic, etc.)	STROBE-AMS
	Dose ***(How much)*** (e.g. 500 mg)	STROBE-AMS
	When was the antimicrobial treatment administered? (e.g. 3 times/day, in the morning, after food)	TIDieR/CONSORT
	Is dose provided as defined daily dosage ***(DDDs)***?	STROBE-AMS
	If not, ***other measurement used*** with justification (e.g. packages, prescriptions)	STROBE-AMS
	Duration of exposure ***(How long)*** (e.g. 7 days)	STROBE-AMS
	Route of administration ***(Mode of delivery)*** (e.g. oral, ointment, etc.)	STROBE-AMS
	Rationale for ***grouping*** of antimicrobials (if applicable)	STROBE-AMS
	Who administered the antimicrobial treatment? (e.g. researcher, clinicians, nurses)	TIDieR
	Was the antimicrobial exposure planned to be ***personalised, or titrated***? (personalised doses by body weight, route specific administration, age, excipient)	TIDieR
	If so, was the what, why, when, and how of it described?	TIDieR
	fidelity – planned: How, and when antimicrobial consumption data were obtained (e.g. pharmacy record, patients' diary to be filled in daily, etc.)	TIDieR
Description of comparator (if applicable)	Type and combinations of antimicrobials ***(What)*** (e.g. Amoxicillin, Amoxicillin-clavulanic, etc.)	STROBE-AMS
	Dose ***(How much)*** (e.g. 500 mg)	STROBE-AMS
	When was the antimicrobial treatment administered? (e.g. 3 times/day, in the morning, after food)	TIDieR/CONSORT
	Is dose provided as defined daily dosage (***DDDs***)?	STROBE-AMS
	If not, ***other measurement used*** with justification (e.g. packages, prescriptions)	STROBE-AMS
	Duration of exposure ***(How long)*** (e.g. 7 days)	STROBE-AMS
	Route of administration ***(Mode of delivery)*** (e.g. Oral, ointment, etc.)	STROBE-AMS
	Rationale for ***grouping*** of antimicrobials (if applicable)	STROBE-AMS
	Who administered the antimicrobial treatment? (e.g. researcher, clinicians, nurses)	TIDieR
	Was the antimicrobial exposure planned to be ***personalised, or titrated***? (personalised doses by body weight, route specific administration, age, excipient)	TIDieR
	If so, was the what, why, when, and how of it described?	TIDieR
	Comparator fidelity – planned: How, and when antimicrobial consumption data were obtained (e.g. pharmacy record, patients' diary to be filled in daily, etc.)	TIDieR
Outcome measures	Defined pre-specified ***primary and secondary*** outcome measures	CONSORT
	When was each outcome measured?	CONSORT
	Definition of ***infection*** or ***colonisation*** used. If new definition, then evidence of robustness of the new definition	STROBE-AMS
	Definition of ***resistance*** (e.g. MIC values, cut-off points), multidrug resistance, and co-resistance used	STROBE-AMS

	Guidelines used in laboratory resistance measurements (NCCLS/CLSI, EUCAST, National German standards, etc.)	Added Item
Sampling: -	***site***	Added Item
	number of samples per person	Added Item
	sampling ***period***	Added Item
	method of sampling (e.g. midstream urine catch)	Added Item
	transport of samples (e.g. transport medium)	Added Item
Sample size	How was ***sample size*** determined?	STROBE/CONSORT
Measurement	Was resistance measured by an independent laboratory?	Added Item
	If not, were microbiologists blinded to exposure arm	Added Item
	Were ***microbiologists*** blinded to the ***time of sampling***?	Added Item
Analysis	***Unit of analysis*** defined (isolates, participants, other)	STROBE-AMS
	Statistical methods used to compare groups for primary and secondary outcomes	STROBE/CONSORT
	Methods for additional analyses, such as ***subgroup analyses*** (e.g., by class of antibiotic exposure) and ***adjusted analyses***	CONSORT/STROBE-AMS
**Results**		
Participants' characteristics and flow	Give ***characteristics*** of study participants (e.g., demographic, clinical, social)	STROBE/CONSORT
	Baseline resistance of the index pathogen	Added Item
	Time since ***last antibiotic exposure***	Added Item
	Description of ***potential confounders***	STROBE/STROBE-AMS
	Information on ***exposures*** (Day care centres for children, and long-term care facilities, nursing home and other healthcare settings for adults)	STROBE-AMS
	Indicate number of participants with ***missing data for each variable*** of interest	STROBE
	Summary of ***follow-up time*** (e.g., average and total amount)	STROBE
Numbers analysed (Exposure)	Incident ***total number*** of participants with/isolates of the ***index pathogen*** at nominated time points	Added Item
	Number of participants not carrying the index pathogen (i.e. ***sterile swabs***) at each time point	Added Item
	Incident number of participants with/isolates of the ***index pathogen*** at nominated time points ***susceptible*** to the ***exposed antimicrobial or same class*** included in each analysis	Added Item
	Incident number of participants with/isolates of the ***index pathogen*** at nominated time points ***resistant*** to the ***exposed antimicrobial or same class*** included in each analysis	Added Item
	Incident number of participants with/isolates of the ***index pathogen*** at nominated time points ***susceptible*** to ***other antimicrobial or different class*** (Co-resistance data) included in each analysis	Added Item
	Incident number of participants with/isolates of the ***index pathogen*** at nominated time points ***resistant*** to ***other antimicrobial or different class*** (Co-resistance data) included in each analysis	Added Item
	Incident number of participants with/isolates of ***other organisms*** at nominated time points ***susceptible*** to the ***exposed antimicrobial or same class*** (from other body sites e.g., bowel, nasopharynx, skin) included in each analysis	Added Item
	Incident number of participants with/isolates of ***other organisms*** at nominated time points ***resistant*** to the exposed antimicrobial or same class (from other body sites e.g., bowel, nasopharynx, skin) included in each analysis	Added Item
	Incident ***total number*** of participants with/isolates of ***other organisms*** at nominated time points (from other body sites e.g., bowel, nasopharynx, skin) included in each analysis	Added Item
	Number of ***adverse events*** occurred during antibiotic treatment reported (nausea, rash, diarrhoea, superinfections, etc.)	CONSORT
Numbers analysed (comparator) if applicable	Incident ***total number*** of participants with/isolates of the ***index pathogen*** at nominated time points	Added Item
	Number of participants not carrying the index pathogen (i.e. ***sterile swabs***) at each time point	Added Item
	Incident number of participants with/isolates of the ***index pathogen*** at nominated time points ***susceptible*** to the ***exposed antimicrobial or same class*** included in each analysis	Added Item

	Incident number of participants with/isolates of the ***index pathogen*** at nominated time points ***resistant*** to the ***exposed antimicrobial or same class*** included in each analysis	Added Item
	Incident number of participants with/isolates of the ***index pathogen*** at nominated time points ***susceptible*** to ***other antimicrobial or different class*** (Co-resistance data) included in each analysis	Added Item
	Incident number of participants with/isolates of the ***index pathogen*** at nominated time points ***resistant*** to ***other antimicrobial or different class*** (Co-resistance data) included in each analysis	Added Item
	Incident number of participants with/isolates of ***other organisms*** at nominated time points ***susceptible*** to the ***exposed antimicrobial or same class*** (from other body sites e.g., bowel, nasopharynx, skin) included in each analysis	Added Item
	Incident number of participants with/isolates of ***other organisms*** at nominated time points ***resistant*** to the ***exposed antimicrobial or same class*** (from other body sites e.g., bowel, nasopharynx, skin) included in each analysis	Added Item
	Incident ***total number*** of participants with/isolates of ***other organisms*** at nominated time points (from other body sites e.g., bowel, nasopharynx, skin) included in each analysis	Added Item
	Number of ***adverse events*** occurred during antibiotic treatment reported (nausea, rash, diarrhoea, superinfections, etc.)	CONSORT
	Comparator fidelity/adherence – actual: The extent to which the antimicrobial was actually used or dispensed (if applicable)	TIDieR
Estimation	For each ***primary and secondary outcome***, and the estimated effect size and its precision (such as 95% confidence interval) (if applicable)	CONSORT
	Results of any ***other analysis*** performed, including ***subgroup analysis*** (by type of patients, type of microorganism, by class of antibiotic exposure)	STROBE/CONSORT/STROBE-AMS
**Discussion**		
	Limitations of the study, taking into account sources of potential bias or imprecision (both direction and magnitude of any potential bias)	STROBE/CONSORT
	Discuss study setting, type of hospital, local epidemiology for ***generalisability*** (external validity) of the study results	STROBE/CONSORT/STROBE-AMS
	Country's resistance pattern	Added Item
	If resistance-related outcomes are different between the comparison groups, discuss the implications for policy and practice	Added Item
	Interpretation consistent with results, balancing benefits and harms, and considering other relevant evidence (if applicable)	CONSORT
Other information	Sources of ***funding*** and other support (such as supply of drugs), role of funders	STROBE/CONSORT
