# Supplementary material for: An analysis of reporting quality of prospective studies examining community antibiotic use and resistance
Source: Trials. 2018 Nov 27;19:656. doi: 10.1186/s13063-018-3040-6 (PMC6258384; doi:10.1186/s13063-018-3040-6)
Supplement: Supplementary file 3 — List of studies included in the systematic review and assessed for their quality. (PDF 122 kb) [file 13063_2018_3040_MOESM3_ESM.pdf]

Additional file 3. List of studies included in the systematic review and assessed for their quality [1-18]

Trials

1. Berg HF, Tjhie JH, Scheffer GJ, Peeters MF, van Keulen PH, Kluytmans JA, Stobberingh EE: **Emergence and persistence of macrolide resistance in oropharyngeal flora and elimination of nasal carriage of Staphylococcus aureus after therapy with slow-release clarithromycin: a randomized, double-blind, placebo-controlled study**. *Antimicrob Agents Chemother* 2004, **48**(11):4183-4188.
2. Chern KC, Shrestha SK, Cevallos V, Dhami HL, Tiwari P, Chern L, Whitcher JP, Lietman TM: **Alterations in the conjunctival bacterial flora following a single dose of azithromycin in a trachoma endemic area**. *Br J Ophthalmol* 1999, **83**(12):1332-1335.
3. Cohen R, Bingen E, Varon E, de La Rocque F, Brahimi N, Levy C, Boucherat M, Langue J, Geslin P: **Change in nasopharyngeal carriage of Streptococcus pneumoniae resulting from antibiotic therapy for acute otitis media in children**. *Pediatr Infect Dis J* 1997, **16**(6):555-560.
4. Cohen R, Navel M, Grunberg J, Boucherat M, Geslin P, Derriennic M, Pichon F, Goehrs JM: **One dose ceftriaxone vs. ten days of amoxicillin/clavulanate therapy for acute otitis media: clinical efficacy and change in nasopharyngeal flora**. *Pediatr Infect Dis J* 1999, **18**(5):403-409.
5. Cremieux AC, Muller-Serieys C, Panhard X, Delatour F, Tchimichkian M, Mentre F, Andremont A: **Emergence of resistance in normal human aerobic commensal flora during telithromycin and amoxicillin-clavulanic acid treatments**. *Antimicrob Agents Chemother* 2003, **47**(6):2030-2035.
6. Dabernat H, Geslin P, Megraud F, Begue P, Boulesteix J, Dubreuil C, de La Roque F, Trinh A, Scheimberg A: **Effects of cefixime or co-amoxiclav treatment on nasopharyngeal carriage of Streptococcus pneumoniae and Haemophilus influenzae in children with acute otitis media**. *The Journal of antimicrobial chemotherapy* 1998, **41**(2):253-258.
7. Eliasson I, Holst E, Molstad S, Kamme C: **Emergence and persistence of beta-lactamase-producing bacteria in the upper respiratory tract in children treated with beta-lactam antibiotics**. *Am J Med* 1990, **88**(5A):51S-55S.
8. Gaynor BD, Chidambaram JD, Cevallos V, Miao Y, Miller K, Jha HC, Bhatta RC, Chaudhary JSP, Holm SO, Whitcher JP *et al*: **Topical ocular antibiotics induce bacterial resistance at extraocular sites**. *British Journal of Ophthalmology* 2005, **89**(9):1097-1099.
9. Ghaffar F, Friedland IR, Katz K, Muniz LS, Smith JL, Davis P, Reynolds J, McCracken GH, Jr.: **Increased carriage of resistant non-pneumococcal alpha-hemolytic streptococci after antibiotic therapy**. *J Pediatr* 1999, **135**(5):618-623.
10. Ghaffar F, Muniz LS, Katz K, Smith JL, Shouse T, Davis P, McCracken GH, Jr.: **Effects of large dosages of amoxicillin/clavulanate or azithromycin on nasopharyngeal carriage of Streptococcus pneumoniae, Haemophilus influenzae, nonpneumococcal alpha-hemolytic streptococci, and Staphylococcus aureus in children with acute otitis media**. *Clin Infect Dis* 2002, **34**(10):1301-1309.**[Same study reported in reference number 9]**
11. Huovinen P, Mattila T, Kiminki O, Pulkkinen L, Huovinen S, Koskela M, Sunila R, Toivanen P: **Emergence of trimethoprim resistance in fecal flora**. *Antimicrob Agents Chemother* 1985, **28**(2):354-356.
12. Malhotra-Kumar S, Lammens C, Coenen S, Van Herck K, Goossens H: **Effect of azithromycin and clarithromycin therapy on pharyngeal carriage of macrolide-resistant streptococci in

healthy volunteers: a randomised, double-blind, placebo-controlled study. *Lancet* 2007, **369**(9560):482-490.
13. Malhotra-Kumar S, Van Heirstraeten L, Coenen S, Lammens C, Adriaenssens N, Kowalczyk A, Godycki-Cwirko M, Bielicka Z, Hupkova H, Lannering C *et al*: **Impact of amoxicillin therapy on resistance selection in patients with community-acquired lower respiratory tract infections: a randomized, placebo-controlled study**. *The Journal of antimicrobial chemotherapy* 2016, **71**(11):3258-3267.
14. Murray BE, Rensimer ER, DuPont HL: **Emergence of high-level trimethoprim resistance in fecal Escherichia coli during oral administration of trimethoprim or trimethoprim--sulfamethoxazole**. *N Engl J Med* 1982, **306**(3):130-135.
15. Nord CE, Peterson J, Ambruzs M, Fisher AC: **Levofloxacin versus azithromycin on the oropharyngeal carriage and selection of antibacterial- resistant streptococci in the microflora of healthy adults**. *Curr Med Res Opin* 2009, **25**(6):1461-1467.
16. Schrag SJ, Pena C, Fernandez J, Sanchez J, Gomez V, Perez E, Feris JM, Besser RE: **Effect of short-course, high-dose amoxicillin therapy on resistant pneumococcal carriage: a randomized trial**. *JAMA* 2001, **286**(1):49-56.
17. Skalet AH, Cevallos V, Ayele B, Gebre T, Zhou Z, Jorgensen JH, Zerihun M, Habte D, Assefa Y, Emerson PM *et al*: **Antibiotic selection pressure and macrolide resistance in nasopharyngeal Streptococcus pneumoniae: a cluster-randomized clinical trial**. *PLoS Med* 2010, **7**(12):e1000377.
18. Toltzis P, Dul M, O'Riordan MA, Toltzis H, Blumer JL: **Impact of amoxicillin on pneumococcal colonization compared with other therapies for acute otitis media**. *Pediatr Infect Dis J* 2005, **24**(1):24-28.

Prospective cohort studies

[1-8]

1. Brook I: **Emergence and persistence of beta-lactamase-producing bacteria in the oropharynx following penicillin treatment**. *Arch Otolaryngol Head Neck Surg* 1988, **114**(6):667-670.
2. Chung A, Perera R, Brueggemann AB, Elamin AE, Harnden A, Mayon-White R, Smith S, Crook DW, Mant D: **Effect of antibiotic prescribing on antibiotic resistance in individual children in primary care: prospective cohort study**. *BMJ* 2007, **335**(7617):429.
3. Conradi AD, Calbo E, Cuchi E, Puig RG, Garcia-Rey C, Boada LT, Diaz-Infantes M, Martin-Herrero JE, Garau J, Spanish Pneumococcal Infection Study N: **Impact of amoxicillin, associated or not with clavulanic acid, on pharyngeal colonization and selection of Streptococcus pneumoniae resistance in children under 5 years of age**. *Eur J Pediatr* 2007, **166**(5):467-471.
4. Dagan R, Leibovitz E, Greenberg D, Yagupsky P, Fliss DM, Leiberman A: **Dynamics of pneumococcal nasopharyngeal colonization during the first days of antibiotic treatment in pediatric patients**. *Pediatr Infect Dis J* 1998, **17**(10):880-885.
5. Lofmark S, Jernberg C, Jansson JK, Edlund C: **Clindamycin-induced enrichment and long-term persistence of resistant Bacteroides spp. and resistance genes**. *The Journal of antimicrobial chemotherapy* 2006, **58**(6):1160-1167.
6. Morita JY, Kahn E, Thompson T, Laclaire L, Beall B, Gherardi G, O'Brien KL, Schwartz B: **Impact of azithromycin on oropharyngeal carriage of group A Streptococcus and nasopharyngeal carriage of macrolide-resistant Streptococcus pneumoniae**. *Pediatr Infect Dis J* 2000, **19**(1):41-46.
7. Raum E, Lietzau S, von Baum H, Marre R, Brenner H: **Changes in Escherichia coli resistance patterns during and after antibiotic therapy: a longitudinal study among outpatients in Germany**. *Clinical Microbiology and Infection* 2008, **14**(1):41-48.
8. Varon E, Levy C, De La Rocque F, Boucherat M, Deforche D, Podglajen I, Navel M, Cohen R: **Impact of antimicrobial therapy on nasopharyngeal carriage of Streptococcus pneumoniae, Haemophilus influenzae, and Branhamella catarrhalis in children with respiratory tract infections**. *Clin Infect Dis* 2000, **31**(2):477-481.
