# Supplementary material for: An analysis of reporting quality of prospective studies examining community antibiotic use and resistance
Source: Trials. 2018 Nov 27;19:656. doi: 10.1186/s13063-018-3040-6 (PMC6258384; doi:10.1186/s13063-018-3040-6)
Supplement: Supplementary file 4 — Quality of reporting, percentage of RCTs meeting each item including the ‘if applicable’ items. (PDF 128 kb) [file 13063_2018_3040_MOESM4_ESM.pdf]

Additional file 4. Quality of reporting, % of RCTs meeting each ***item*** (studies= 17, including if applicable items)

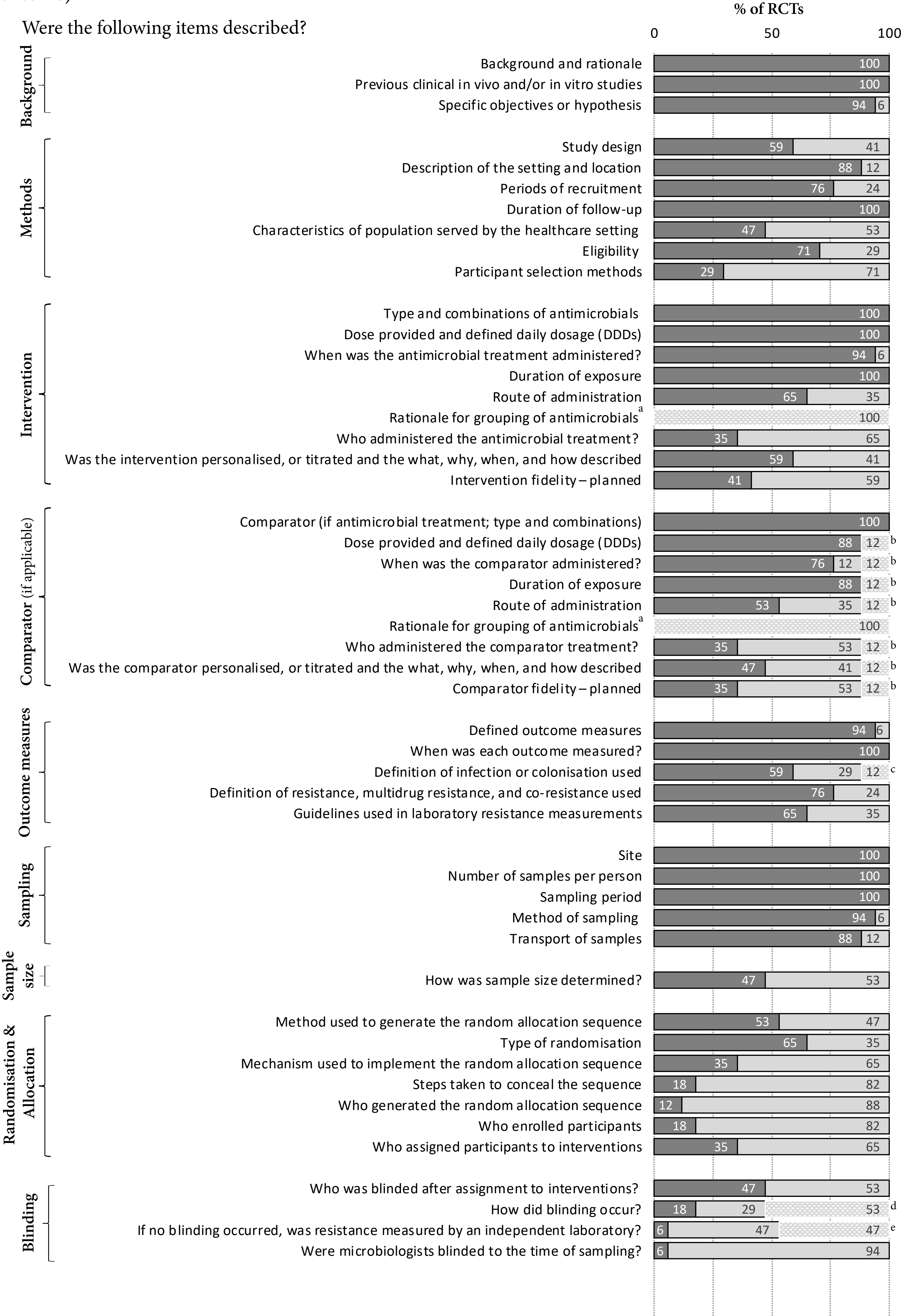

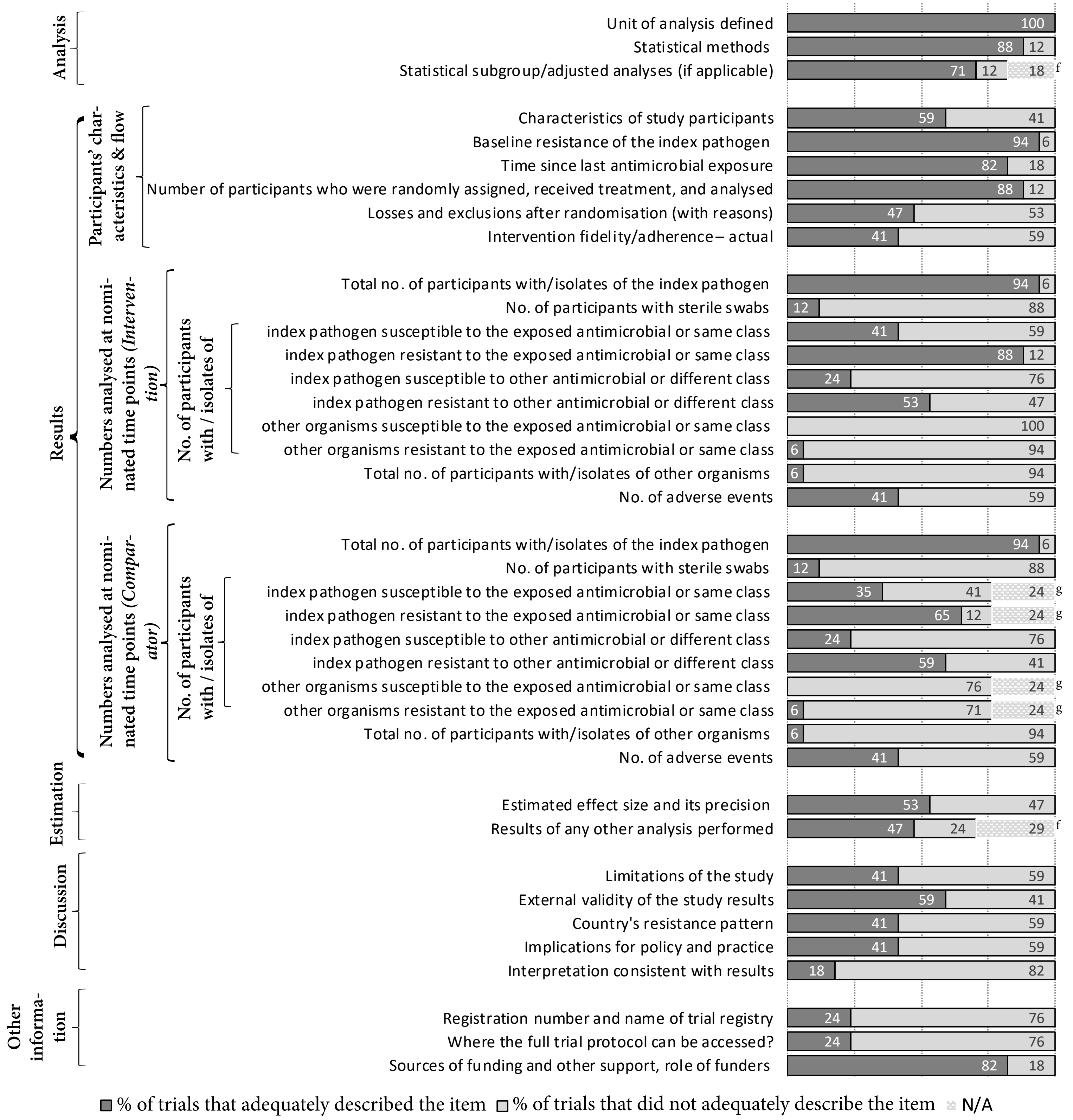

[a] Not applicable because it was not within the scope of the main review
[b] Not applicable for studies that compared the intervention to a no-exposure (control) group
[c] Not applicable for studies that recruited healthy participants
[d] Only applicable for blinded studies
[e] Only applicable for studies where no blinding occured
[f] Only applicable for studies that reported subgroup/adjusted analyses
[g] Not applicable for studies that compared the intervention to a control/placebo group
