# Supplementary material for: An analysis of reporting quality of prospective studies examining community antibiotic use and resistance
Source: Trials. 2018 Nov 27;19:656. doi: 10.1186/s13063-018-3040-6 (PMC6258384; doi:10.1186/s13063-018-3040-6)

Additional file 5. Quality of reporting, % of items described by each trial (studies= 17, mandatory items= 70)

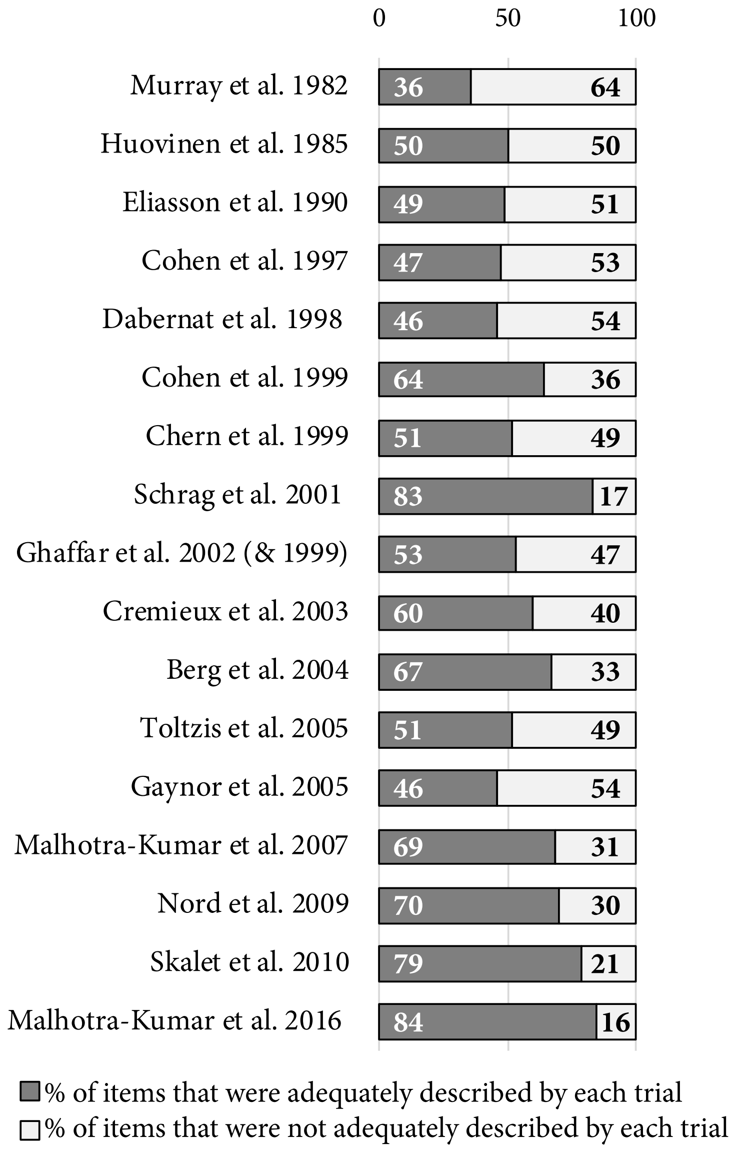

Supplement: Supplementary file 5 — Quality of reporting, percentage of items described by each trial. (PDF 29 kb) [file 13063_2018_3040_MOESM5_ESM.pdf]
