# Supplementary material for: An analysis of reporting quality of prospective studies examining community antibiotic use and resistance
Source: Trials. 2018 Nov 27;19:656. doi: 10.1186/s13063-018-3040-6 (PMC6258384; doi:10.1186/s13063-018-3040-6)
Supplement: Supplementary file 6 — Quality of reporting, percentage of cohort studies meeting each item including the ‘if applicable’ items. (PDF 127 kb) [file 13063_2018_3040_MOESM6_ESM.pdf]

Additional file 6. Quality of reporting, % of cohort studies meeting each *item* (studies= 8, including if applicable items)

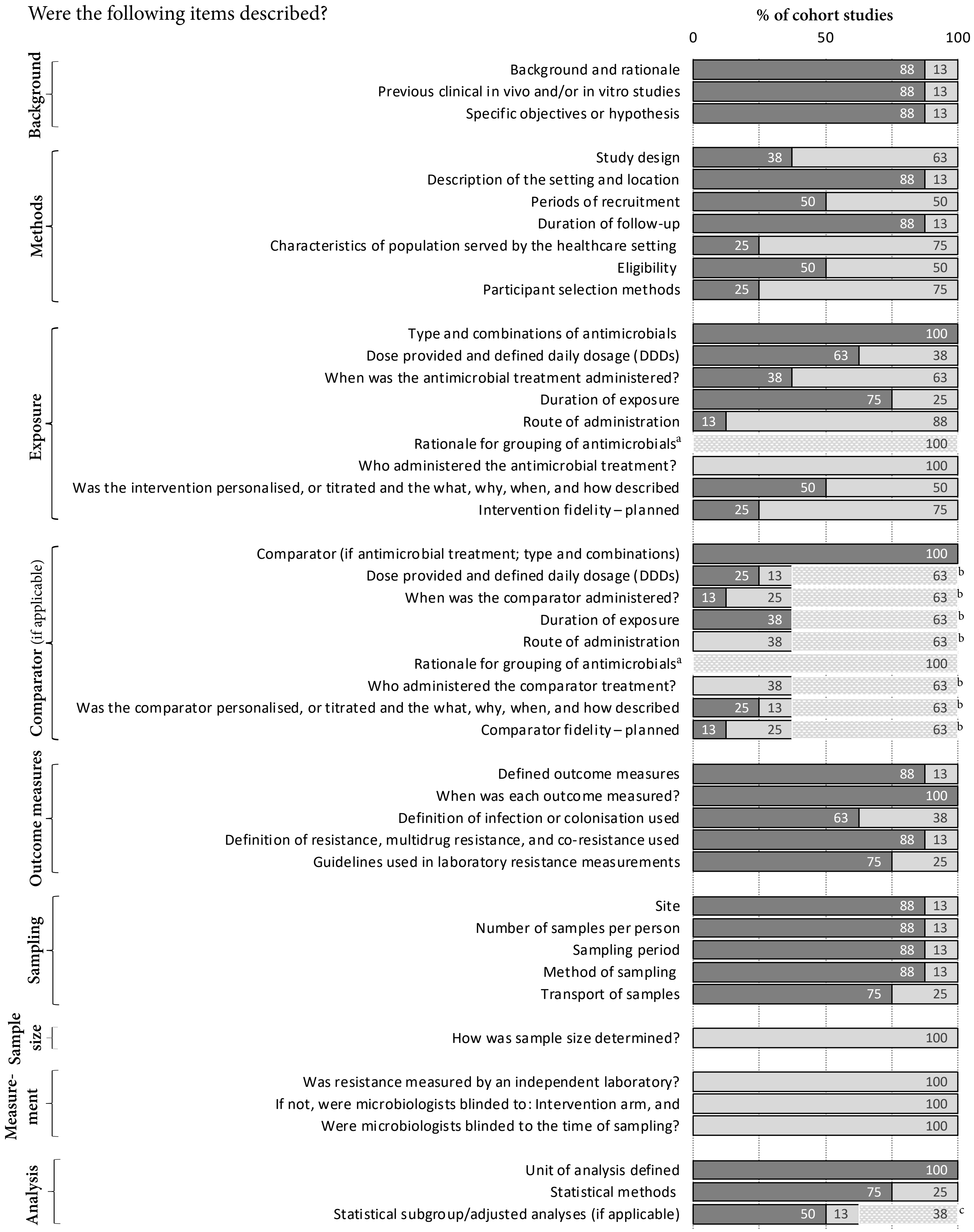

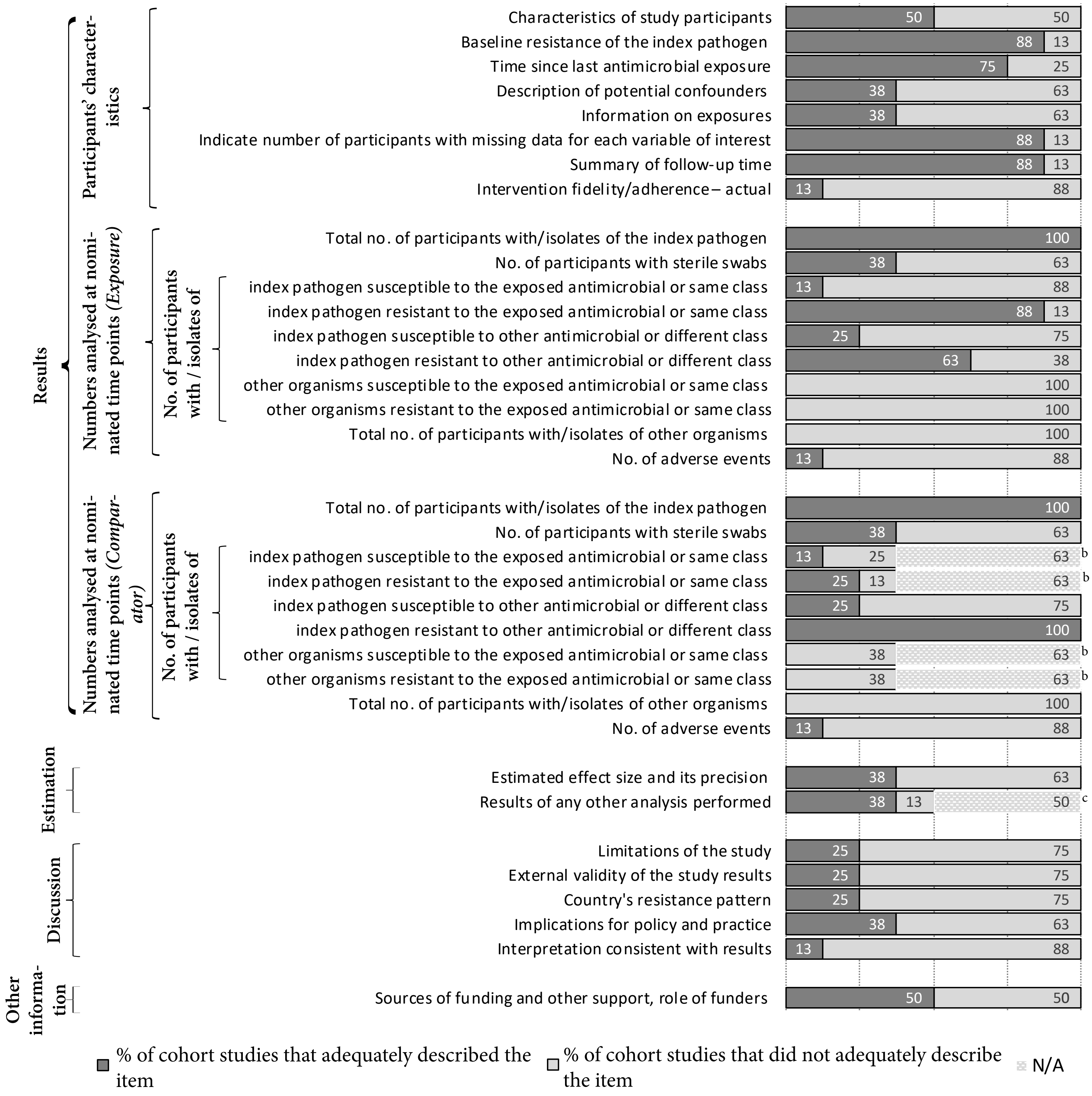

[a] Not applicable because it was not within the scope of the review
[b] Not applicable for studies that compared the intervention to a no-exposure (control) group
[c] Only applicable for studies that reported subgroup/adjusted analyses
