# Supplementary material for: An analysis of reporting quality of prospective studies examining community antibiotic use and resistance
Source: Trials. 2018 Nov 27;19:656. doi: 10.1186/s13063-018-3040-6 (PMC6258384; doi:10.1186/s13063-018-3040-6)

Additional file 7. Quality of reporting, % of items described by each ***cohort*** study (studies= 8, mandatory items= 63)

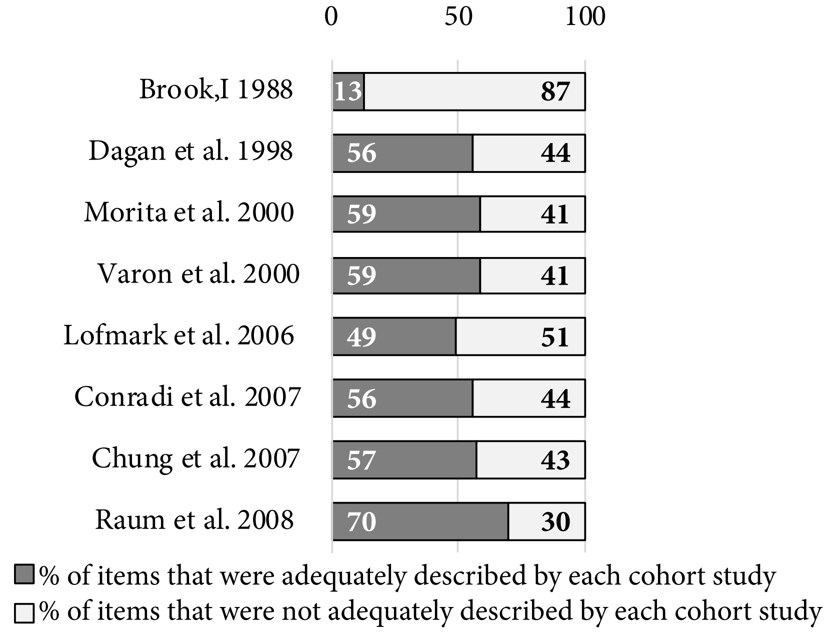

Supplement: Supplementary file 7 — Quality of reporting, percentage of items described by each cohort study. (PDF 31 kb) [file 13063_2018_3040_MOESM7_ESM.pdf]
